# Neurogenesis and the Epigenetic Landscape: Role of Histone Modifications and Chromatin Remodeling

**DOI:** 10.1002/brb3.71223

**Published:** 2026-01-28

**Authors:** Degisew Yinur Mengistu, Biniam Moges Eskeziyaw

**Affiliations:** ^1^ Department of Biology and Biotechnology “Charles Darwin” Sapienza University Rome Italy; ^2^ Institute of Biotechnology University of Gondar Gondar Ethiopia; ^3^ Department of Biotechnology Debre Berhan University Debre Berhan Ethiopia

**Keywords:** Chromatin remodeling, histone deacetylases, histone methylation, neural stem cell, proliferation, symmetric division

## Abstract

**Aims:**

The purpose of this review is to examine how epigenetic regulation particularly chromatin modification and histone methylation controls gene expression during embryonic neurogenesis. It aims to highlight the role of these mechanisms in neural stem cell (NSC) fate specification and their implications in neurological and neurodevelopmental disorders.

**Methods:**

Through reviewing recent research findings, this study synthesizes current literature on epigenetic mechanisms involved in embryonic brain development, with a focus on histone modifications, chromatin remodeling, and chromatin compartmentalization. The review also evaluates existing in vivo research while noting the technical challenges of tracking adult neurons and isolating NSCs.

**Findings:**

The review identifies that epigenetic mechanisms, including histone methylation (notably H3K9 as a repressive mark), histone deacetylases, and chromatin remodeling complexes, play essential roles in regulating gene expression required for neurogenesis and neuroplasticity. Alterations in these epigenetic processes significantly affect neural development and contribute to a range of neurological and neurodevelopmental disorders.

**Conclusions:**

Understanding the epigenetic regulation of neurogenesis particularly through chromatin modification and structural chromatin dynamics provides valuable insight into cell fate determination during embryonic brain development. These insights may guide the development of novel therapeutic strategies for neurological and neurodevelopmental disorders.

## Graphical abstract

Histone methylation and acetylation modulate gene expression by inducing chromatin condensation or relaxation, which in turn regulates transcriptional activity and impacts neurogenesis.

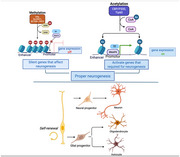

## 1 Introduction

Neural stem cells (NSCs) division during the developmental process directly determines the neuron numbers in the adult brain. Regulation of NSC neurogenesis occurs in both insects and mammals through a sequence of self‐renewing divisions (Mora et al. 2018). The number of neurons is mainly determined by the proliferation potential of neural stem and progenitor cells (NSPCs), which is achieved by the lineage NSPCs, the number of symmetric and asymmetric divisions, and by the cell cycle frequency (Homem et al. 2015; Zahr et al. 2019). During asymmetric division of NSCs, NSCs self‐renew and generate daughter cells with different proliferation potential. The patterns of asymmetric division of NSCs are classified into three different broad categories: multiple dividing daughter cells, nondividing daughter cells, and once‐dividing daughter cells (Taverna et al. 2014; Zahr et al. 2019). On the other hand, in symmetric division of NSCs, there are only two division patterns. One type is the end of stem cells by generating of two daughter cells that commit to differentiation, and the second, which occurs rarely, expands the progenitor pool by producing two cells (Mora et al. 2018; Marthiens and Basto 2020).

Various physiological and environmental stimuli affect the regulation of neurogenesis. Recognition of the morphology and expression markers of NSCs and their progeny improves understanding of how NSCs are regulated by various stimuli and molecular and genetic bases. Particularly, understanding how NSC self‐renewal and neuron generation both in vivo and in vitro may provide new insights and lead to foster breakthroughs to transform basic research into clinical trials (Akers et al. 2018). However, studies in vivo remain limited because of the difficulty in tracking adult neurons in vivo as well as isolating and detecting NSCs in vitro. Additionally, the mechanism of how the division pattern of NSCs changes over time and whether this alteration leads to the formation of the correct neuron number is also still unclear.

Epigenetic mechanisms such as posttranslational histone modifications (HPTMs), DNA methylation, chromatin remodeling, and regulation mediated by noncoding RNAs result changing of the function of genetic elements orthe pattern of gene expression (Zoghbi and Beaudet 2016; Jih et al. 2017; Albert and Huttner 2018). These epigenetic signals regulate the cell's transition to differentiation or the maintenance of its self‐renewal. During neurogenesis and neuroplasticity, histone methylation and dynamic DNA modification play a pivotal role. Recent studies reveal that abnormal histone methylation and acetylation are linked to neurological disorder and threaten human health (Mora et al. 2018). The lysine (Lys, K) residue, which occurs primarily on histone H3 and H4, is the most common acceptor site for histone methylation (M. Zhang et al. 2020). Like H3K9 methylation, H3K9 acetylation is directly or indirectly correlated with transcriptomic changes related to neurogenesis (Shakèd et al. 2008; MacDonald and Roskams 2008). Moreover, the three‐dimensional (3D) architecture of chromatin exhibits dynamic changes during neurogenesis, suggesting an important role of chromatin arrangement in cell fate determination (Bonev et al. 2017).

In this review, we focus on the epigenetic aspect of H3K9 methylation, analyzing insights gained from studies of neurodevelopment and neurogenesis by process through symmetric and asymmetric division. We then address how chromatin compartmentalization affects genome stability, neurogenesis, and its role in neurodevelopmental disease.

## 2 Mammalian Neural Progenitors

During embryo development, neurogenesis is ensured by NSPCs in the lateral ganglionic eminence (LGE) and persists in the adult and aged subventricular zone (SVZ) (Ahlenius et al. 2009). The specialized epithelium, lining the lumen of the lateral ventricle called neuroepithelium, is the source of all neurons in mammals (Farkas and Huttner 2008). A small number of NSPCs generate neurons in the brain. During early gestation, neuroepithelial cells (NECs) initially undergo symmetric divisions to expand the progenitor pool, and later transition to asymmetric divisions that give rise to apical radial glial cells (aRGs) in the developing mammalian brain. The aRGs first divide symmetrically within the ventricular zone (VZ) to generate additional aRGs (Mira and Morante 2020). Subsequently, they shift toward neurogenic divisions, producing neurons either directly through asymmetric division that yields one self‐renewing aRG and one neuron or indirectly by generating intermediate neural progenitors (INPs) capable of further proliferation, thereby amplifying neuronal output (Cárdenas and Borrell 2019).

All the types of cells in the brain such as neuronal cells, astrocytes, and oligodendrocytes (OLs) can be generated from NSPCs (Goldman and Luskin 1998; Gage 2000; Mignone et al. 2004; Gao et al. 2014). The initial number of NSPCs and their proliferation during neurogenesis are not the sole determinant of the number of neurons in the brain. The number of neurons is also determined by the lineage of NSPCs (Lanet et al. 2013). During proliferation, NSPCs undergo self‐renewal and generate differentiated daughter cells (Ming and Song 2011; Homem et al. 2015). In the developmental process, NSPCs expand by self‐renewal, whereas they undergo apoptosis or senescence upon completion of neurogenesis. As a result, the number of NSC cells becomes lower after the embryo, and only a few are present in the adult brain. The proliferative potential of NSPCs, the NSPC lineage, the number of symmetric and asymmetric divisions, as well as the cell cycle frequency determine the number of neurons in the brain (Homem et al. 2015).

## 3 Neural Progenitors in *Drosophila*

NSPCs in *Drosophila* brain development are known as neuroblasts (NBs), round shaped stem cells that arise from epithelial cells by delamination, lack of adherents junctions and crumbs, and comprise two types of lineages, type I and type II (Felsenfeld 2014). The *Drosophila* type II NB resembles mammalian NSCs in many routes; *Drosophila* is an important model system for the study of mammalian neural diseases because it is highly analogous to mammalian NSC lineages. After mitosis, each *Drosophila* daughter cell inherits a set of determinants. During asymmetric division of the type II NB, another NB (self‐renewal) and an INP cell are generated. This INP cell then matures and undergoes a limited number of divisions to generate additional INP cells and cells called ganglion mother cells. The process by which stem cells and INP cells become specific cell types is known as differentiation. Nevertheless, under certain circumstances, INPs can undergo dedifferentiation, an opposite process in which they become ectopic NBs, which may lead to excessive cell proliferation (Koe et al. 2014). INPs have spatial features; they undergo maturation within 4–6 h and do not undergo cell division until a mature INP (mINP) has emerged. Immature INPs undergo maturation when both Brain tumor (Brat) and the Notch antagonist Numb function cooperatively (Bowman et al. 2008). Hence, Numb inhibits Notch signaling, which is relevant for the fate of type II NB. The mINP undergoes asymmetric divisions to self‐renew and to produce multiple GMCs (Weng et al. 2010), whereas the type I NB undergoes cell division to self‐renew and generate a ganglion mother cell, which divides one more time to produce two glial cells (Koe et al. 2014). Compared with the type II, the type I lineage generates few glial mother cells. Moreover, in the embryonic ventral nerve cord, the invertebrate counterpart of the vertebrate spinal cord, most NBs initially divide in the type I mode, but later transition to the type 0 mode, in which each NB undergoes repeated asymmetric divisions to generate progeny that differentiate directly into neurons. In contrast, within the tip of the outer proliferation center (t‐OPC), larval NBs switch from type 0 to type I mode, enabling the production of diverse neuronal and glial cell types in the adult optic lobe (Bertet et al. 2014; Mira and Morante 2020).

The apical localized proteins, such as Numb and Miranda, determine the asymmetric division in both type I and type II lineages (Koe et al. 2014). The transcription factor Ase is absent in the type II lineage, and the type II is mainly characterized by the presence of the transcription factor Pointed and Button head (BTD). Type I is less sensitive to Notch signaling, but when Notch levels are reduced, type I NBs decrease in the central brain rather than in the nerve cord (Almeida and Bray 2005). Therefore, Notch signaling plays an important role in maintaining of NB identity and its overactivation leads to dedifferentiation of INPs. This reveals the nature of lineage in which the fates of NB affects the number of neurons in the brain.

## 4 Symmetric/Asymmetric Division of NPSC and Proliferation Control of Neural Stem Cells

Two different daughter cells produced during cell division is called asymmetric cell division. This type of division is common in invertebrates to generate various cell types as well as to balance the competing demands of stem cells through self‐renewal and differentiation (Zhong and Chia 2008; Prehoda 2009). Many cell lineages such as blood, skin, muscle, intestine, mammary gland, and neurons exhibit asymmetric division (Yoo and Kwon 2015). During the development of the vertebrate nervous system, progenitor cells exhibit four principal modes of division. In symmetric proliferative divisions, both daughter cells retain progenitor identity, as seen when an NEC divides to produce two NECs. In contrast, symmetric differentiative divisions yield two differentiated neurons, such as when a basal progenitor generates a pair of neurons. Asymmetric self‐renewing (mono‐differentiative) divisions result in one progenitor and one differentiated neuron, maintaining the progenitor pool while contributing to neurogenesis. Finally, asymmetric bi‐differentiative divisions produce two distinct differentiated cell types, for example, an NEC giving rise to a radial glial cell and a neuron. These division patterns collectively orchestrate the balance between progenitor maintenance and neuronal differentiation during brain development (Huttner and Kosodo 2005).

In mammals and *Drosophila*, NSCs undergo repeated asymmetric division, giving rise to one daughter cell that remains in the stem cell lineage while the other undergoes a limited number of rounds of differentiation and proliferation (Zhong and Chia 2008). The fate of cell types, whether daughter glial/neurons or stem cells, is determined by the type of cell division performed by NSCs.

In *Drosophila*, neurons are generated by embryonic and larval NBs through repeated asymmetric divisions. NBs originally formed at the embryonic stage produce only 10% of adult neurons. In contrast, larval NBs generate 90% of adult neurons (Prehoda 2009; Lim et al. 2017). After division, larval NBs replace their cytoplasm, whereas embryonic variants divide rapidly and shrink during embryogenesis (Ito and Hotta 1992).

A recent study has shown that a subclass of larval central brain NBs exhibits a mode of neurogenesis, which remarkably resembles that observed in the mammalian central nervous system (CNS), where the primary progenitors amplify the progeny number produced through the generation of proliferating intermediate progenitors (Bello et al. 2008).

The architecture of cellular symmetry and asymmetry is supported and regulated by polarity proteins and cell–cell adhesion proteins. In particular, a family of well‐conserved proteins essential for mitotic spindle orientation, termed polarity proteins, has been identified as key regulators of symmetric and asymmetric division (Suzuki and Ohno 2006). The fate of the transition between symmetric and asymmetric division must be precisely regulated because it determines brain cell size. For example, excessive tumor growth is caused by excessive symmetric divisions, whereas premature differentiation and small brains are the result of premature asymmetric division (Cabernard and Doe 2009).

Key proteins that play a critical role in NB polarity are known. The two evolutionarily conserved protein complexes are the Par proteins (homologs of Par3/Par6/aPKC), a protein cassette related to heterotrimeric G protein signaling (Gai/Pins/Loco), as well as Inscuteable protein (Insc), which can bind both Par3 and Pins (Zhong and Chia 2008). Par3/Bazooka (Baz) is essential for polarization of the embryonic ectoderm, the first epithelium formed immediately before gastrulation by a unique process called cellularization. Polarity is initially defined by Par protein, which is located at the apical end of the neuroepithelium, aligns along the apical basal axis during NB delamination. Then, Insc and the G protein cassette are recruited to the apical cortex. The asymmetric localization of the cell fate to the basal cortex (opposite) and the orientation of the mitotic spindle are determined by the Par protein and the G protein‐related cassette, respectively (Knoblich 2008).

**FIGURE 1:**
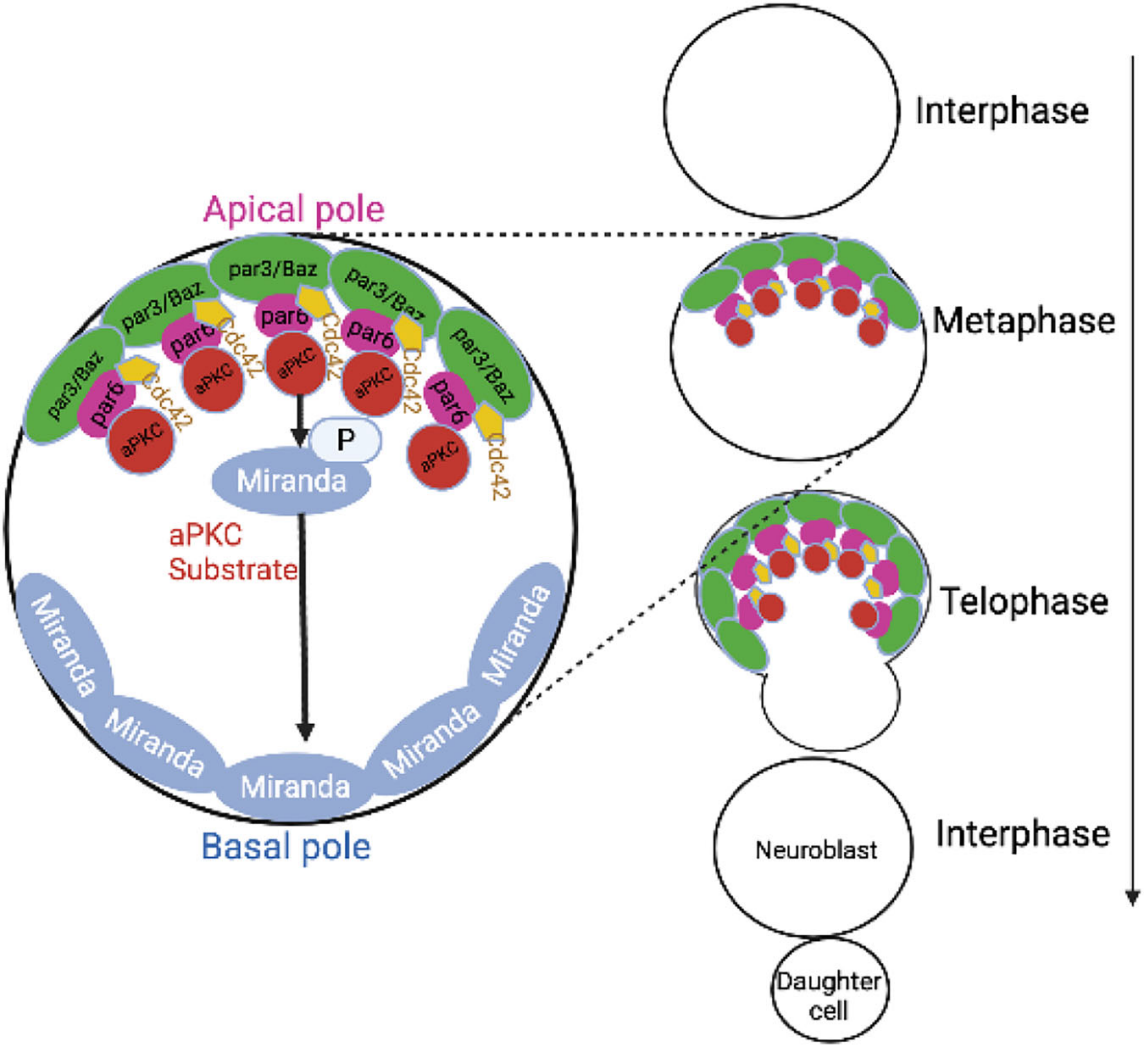
*Drosophila* Baz (Par‐3 homolog) localizes with the Cdc42‐Par6‐aPKC complex to the apical pole of NB at mitosis. Clustering of complexes via oligomeric interactions is crucial for the formation of a polarized apical plasma membrane domain (modified from Thompson [2022]).

The polarization of Baz to the apical pole of the NB does not occur until mitosis. During early prometaphase, the localization of Baz to the apical pole is elicited by Aurora A cell cycle kinase. The Ap protein, which is evolutionarily novel and plays a crucial role in apical recruitment of Baz during mitosis, is called Inscuteable protein (Insc) (Knoblich 2008; Guerra et al. 2022). In general, in order to polarize the cell during metaphase, Par protein moves forward to the apical cortex of the NB, whereas Miranda localizes to the basal pole; an intact actin cytoskeleton is required for this process (Figure 1). Proper orientation of the mitotic spindle is critical for asymmetric segregation of cell fate determinants (Bello et al. 2008; Felsenfeld 2014).

As in *Drosophila*, there is a precise correlation between spindle orientation and the asymmetric outcome of mammalian brain progenitor cell divisions. Intriguingly, the pathways involved are conserved and similar in mammals, where they establish radial glial cells as the primary NSCs, functionally analogous to *Drosophila* NBs (Götz and Huttner 2005). For instance, heterotrimeric G proteins and their binding partners Pins and Inscuteable protein are essential for spindle orientation (Konno et al. 2008). A study demonstrated that the evolutionarily conserved Notch signaling pathway and the cell polarity protein Mammalian Par3 (mPar3, also called ASIP in vertebrates) together regulate asymmetric cell division of radial glial progenitor (RGP) cells in the developing neocortex (Bultje et al. 2009). The distribution of mPar3 is not restricted to the apical membrane domain of radial glial cells; rather, it is dynamic and depends on cell cycle progression. This dynamic nature of mPar3 results in its asymmetric inheritance by the two daughter cells, leading to differential activation of Notch signaling. High Notch signaling activity occurs in the daughter cell that inherits a high amount of mPar3 and remains a radial glial cell, whereas the daughter cell that inherits less mPar3 exhibits low Notch signaling activity and adopts either a neuronal or an IPC fate (Bultje et al. 2009). This suggests that the mode of division of radial glial cells may be significantly determined by the subcellular distribution of mPar3 (Bultje et al. 2009).

## 5 Histone Posttranslational Modifications in Neurogenesis

Epigenetic mechanisms, including DNA methylation, histone modifications, chromatin remodeling, and regulation mediated by noncoding RNAs, result in meiotically and mitotically heritable changes in the function of genetic elements or in the pattern of gene expression (Felsenfeld 2014; Guerra et al. 2022). Sets of enzymes regulate epigenetic modifications, and enzymes serve as “erasers” or “writers” to remove or add epigenetic markers, respectively, whereas “readers” bind to these modifications and serve as effectors (Yao et al. 2016). Epigenetic mechanisms are considered equally important in regulating gene expression as sequence‐specific DNA‐binding transcription factors. Among the epigenetic mechanisms, chromatin remodeling is highly correlated with controling of neural cell fate and neurogenesis.

DNA is organized by the formation of nucleosomes into chromatin, which comprises a histone octamer wrapped by roughly 146 base pairs of DNA (Åstrand 2006; X. Li, Egervari et al. 2018). The octamer histone consists of a tetramer of two heterodimers of histone proteins H3/H4 that interacts with two heterodimers of histones H2A/H2B. These histone variants are deposited on the chromatin by specific histone chaperones, which are mainly involved in packaging the DNA into the tiny space called the cell nucleus. In addition, they are posttranslationally modified at the N‐terminal tail to regulate the accessibility of the associated DNA regulatory elements (Adam and Harwell 2020). The action of chromatin modification can occur through two potential mechanisms; the first one is based on the electrostatic interaction between the positively charged N terminus and the negatively charged DNA, and the second on the creation of binding sites for adaptor proteins and transcription factors that specifically recognize modified histone residues (Zentner and Henikoff 2013; Mitrousis et al. 2015). These modifications result in the activation or repression of gene expression. Among histone protein variants, the H3 and H4 families are involved in the regulation of developmental processes and disease, considerably increasing the research interest in the role of HPTMs in neurogenesis (Prehoda 2009; M. Zhang et al. 2020). Indeed, modification on histone 3 at lysine (H3K) methylation plays a pivotal role in both transcriptional activation and silencing activity, mediated by methylation at H3K4, H3K36, and H3K79, or methylation at H3K9 and H3K27 positions, respectively (Bannister and Kouzarides 2011).

## 6 Histone Methylation and Acetylation in Neurogenesis and Genome Stability

The role of H3K9 methylation in genome stability has been the subject of numerous studies showing that DNA damage in heterochromatin and mitotic defects in *Drosophila* are caused by loss of H3K9 methylation (Peng and Karpen 2009). In addition, studies in mice have shown that loss of H3K9me2/me3 leads to disruption of heterochromatin and increases telomere length (Benetti et al. 2007). In human brain cells, the repressive marker H3K9me3, which is distributed in pericentromeric heterochromatin (PCH) is also associated with pericentric satellite repeat sequences (Mansuroglu et al. 2016; Calderón‐Garcidueñas et al. 2020; Thamban et al. 2020), whereas H3K9me2 is not colocalized with chromocenters but is rather enriched at minor satellites of centromeric regions (Allis and Jenuwein 2016; Zheng et al. 2019; Calderón‐Garcidueñas et al. 2020). This implies that H3K9me2/me3 is required to ensure fidelity of lineage specificity and inactivation of developmental stage‐ and tissue‐specific coding genes in heterochromatic regions (Bessler et al. 2010; Calderón‐Garcidueñas et al. 2020). Although the methyl modifications of H3K9me2 and H3K9me3 are located at the same amino acid residues, the localization patterns of the two heterochromatin markers are completely different in the *Caenorhabditis elegans* germline (Bessler et al. 2010).

H3K9 methylation is also involved in the pluripotency of embryonic stem cells (ESCs) and the multipotency of neural precursor cells (NPCs) (Schaefer et al. 2009). However, a universal association between histone methylation and specific histone modifications in the regulation of gene expression has not yet been ascertained (Mitrousis et al. 2015). For instance, increased H3K4me2 and decreased H3K27me3 expression resulted in increased expression of some genes, whereas this was not the case for other genes (Bessler et al. 2010). In *C. elegans* germline cells, the H3K9me2 and H3K9me3 modifications have a different localization pattern and act independently. Moreover, they are associated with different chromatin domains displaying distinct cytological and functional properties; nevertheless, both markers are considered repressive modifications and occur at the same residue of H3 histone (Benetti et al. 2007; Greenberg and Bourc'his 2019). Previous studies showed that H3K9me3 corresponds to the presence of constitutive heterochromatin, while H3K9me2 is enriched on silenced euchromatin (Peters et al. 2003; Shi and Dawe 2006; Bessler et al. 2010).

H3K9 marks may have a potential effect on epigenetic heritability that leads to DNA damage and cell death in addition to their mechanism of silencing of genes (Jih et al. 2017). Treatment of *C. elegans* with G9a HMT inhibitors that contribute to H3K9 demethylation can lead to cell death (Padeken et al. 2019). met‐2 and set‐25 mutants that catalyze H3K9me1 and H3K9me2, as well as the catalytic domain of me1, me2, and me3, show germline lethality and stochastic delays in development (Zeller et al. 2016). This reveals that H3K9 methylation may preclude an essential role in somatic tissue differentiation (Padeken et al. 2019). Another study conducted in Alzheimer's disease (AD) patients and animal models found that in AD patients and animal models, there was a global decrease in the repressive HPTMs H3K9me2 and H3K9me3, as well as lower H3K9me2/me3 and higher γ‐H2AX staining (Calderón‐Garcidueñas et al. 2020). Notably, Calderón‐Garcidueñas et al. (2020) demonstrated these effects specifically in brains exposed to particulate urban air pollution, highlighting the environmental effect associated with H3K9 modifications which result neurodegeneration. In *Drosophila*, oxidative stress can cause DNA damage and reduce H3K9me2 levels (Frost et al. 2014). Loss of H3K9me2 andheterochromatin protein 1α (HP1α) leads to tau‐mediated neurodegeneration in insect and vertebrate systems; tau is a causal factor in the pathogenesis of neurodegenerative diseases (Frost et al. 2014). Therefore, there is emerging evidence for di‐/trimethylation of H3K9, which may play a role in double‐strand break repair (Pappa et al. 2019). All taken together, H3K9me2/me3 heterochromatin markers play a significant role in genome stability and neurogenesis, especially in neurodegenerative diseases.

The histone methyltransferase G9a, which mediates H3K9 methylation, has been shown to negatively regulate memory formation and synaptic plasticity. Notably, pharmacological inhibition of G9a in mouse models of AD alleviated cognitive deficits and restored synaptic function, underscoring the therapeutic potential of targeting this pathway in age‐related neurodegeneration (Tan et al. 2012).

H3K9 functions as a dual regulatory epigenetic mark; depending on the genomic context, it can undergo either methylation or acetylation, resulting in silencing or activation of gene transcription. Methylation of H3K9, especially H3K9me2/me3, is a hallmark of constitutive heterochromatin, where it mediates transcriptional repression, genome stability, and long‐term gene silencing through the recruitment of reader proteins such as HP1. In contrast, acetylation of H3K9 (H3K9ac) is enriched at enhancer elements and particularly at gene promoters, where it presumably facilitates transcription factor binding and RNA polymerase II recruitment by neutralizing the positive charge of lysine residues, thereby promoting chromatin accessibility (Bannister and Kouzarides 2011). The two modifications at the H3K9 residue are mutually independent, enabling H3K9 to serve as a molecular switch that dynamically toggles chromatin between permissive and repressive states. This duality is further reinforced by the presence of H3K9 acetylation and methylation within distinct regulatory regions of the genome, including bivalent chromatin domains, where context‐dependent interpretation by specific reader complexes allows fine‐tuned regulation of gene expression during development and differentiation. This reversible and region‐specific control of H3K9 modifications is central to epigenetic plasticity in processes such as neurogenesis, where rapid transitions between progenitor maintenance and lineage commitment are required (Bannister and Kouzarides 2011). Highlighting this contrast would strengthen the discussion and underscore the dual regulatory potential of the same lysine residue depending on its modification state, with implications for both neural development and genomic integrity (Večeřa et al. 2018). Furthermore, studies have demonstrated the specific impact of certain combinations of histone modifications in the regulation of transcription. For instance, in ESCs, promoters of several development‐associated genes are occupied by H3K4me3 (an active histone mark) and H3K27me3 (a repressive histone mark) to create bivalent domains (Bernstein et al. 2006). This presumably plays a role in maintaining pluripotency by silencing developmental genes in ES cells while keeping them poised for activation (Bernstein et al. 2006). Similarly, another bivalent domain of H3, the K4me3‐K9me3/2 methylation pattern, has been identified as regulating gene expression at low levels, such as in differentiation master genes (Zhao et al. 2020). This implies that histone H3K9me3/2 is not solely enriched in inactive gene regions but also co‐occurs with H3K4me2 in active regions of rDNA. Zhao et al. (2020) characterized a potent H3 “K4me3‐K9me3/2” bivalent mark reader known as Spindlin1, suggesting that the combinational readout of K9me3/2 and K4me3 by Spindlin1 potentially helps to balance gene expression during neurogenesis.

## 7 Chromatin Remodeling Complexes

Flemming (1882) was the first to discover the unique fibrous structures in cellular nuclei, known as chromatin, which is a highly dynamic structure that orchestrates genome organization and thereby regulates gene expression (Mossink et al. 2021). Distinct chromatin structures, including open euchromatin and condensed heterochromatin, were first described by Heitz (1928). The dynamics of chromatin patterns can be altered by chromatin remodelers through various mechanisms, including changes in the conformation of nucleosomal DNA, nucleosome sliding, and histone variant exchange (altering the composition of the octamers) (Mossink et al. 2021). According to their function, chromatin remodelers are categorized as those involved in DNA modifications (which can attractor repel chromatin complexes), enzymes responsible for altering histone posttranslational modifications, and enzymes involved in controlling altered histone–DNA contacts within the nucleosome via ATP hydrolysis (Clapier and Cairns 2009; Mossink et al. 2021). Alteration of genome architecture by chromatin remodeling facilitates downstream gene expression and influences cell proliferation, cell fate, and cellular demand‐specific responses during neurogenesis.

## 8 The Polycomb Group (PRC) Pathway

The Polycomb repressive system demonstrates how developmental processes are controlled by chromatin modification and highlights the significance of chromatin‐based gene regulation in animals (Blackledge and Klose 2021). Polycomb group (PcG) proteins were initially discovered in *Drosophila melanogaster* and are conserved in other animals, including mammals. The Polycomb repressive system comprises two central protein complexes: Polycomb repressive complex 1 (PRC1) and PRC2, both of which posttranslationally modify histones (Piunti and Shilatifard 2021; Blackledge and Klose 2021). PRC1 is a histone ubiquitin ligase responsible for monoubiquitylating histone H2A at Lys119 (H2AK119ub1) (H. Wang et al. 2004; Niekamp et al. 2024), whereas PRC2 is a histone methyltransferase that mono‐, di‐, and trimethylates histone H3 at Lys27; the facultative heterochromatin mark H3K27me3 is abundant in genomic regions (Cao et al. 2002). PRC2 consists of three subunits: enhancer of zeste 2 (EZH2), suppressor of zeste 12 (SUZ12), and embryonic ectoderm development (EED), all of which are essential for proper catalytic activity and transcriptional repression (Di Croce and Helin 2013). Ablation of any of these PRC2 subunits impairs the PRC2 complex, resulting in loss of H3K27me3, aberrant embryonic development, and early embryonic lethality (Montgomery et al. 2005; Leeb and Wutz 2007; Di Croce and Helin 2013). Recent findings have shown that PRC2 regulates temporal RGP lineage progression, and loss of PRC2 activity results neurogenesis defects and microcephaly (Amberg et al. 2022). This study deployed mosaic analysis with double markers (MADM) technology to assess the role of PRC2 at the individual RGP cell level, and found that cell‐autonomous, distinct sequential, and global tissue‐wide functions of PRC2 are essential for RGP lineage progression during cortical development (Amberg et al. 2022). Another study revealed that PRC2 is specifically required for determining glial cell fate choices and the timing of OL lineage progression by repressing the Notch signaling pathway (W. Wang, Cho et al., 2020). Knocking out Eed in the developing mouse dorsal telencephalon results in smaller forebrain/cerebral cortex structures, particularly affecting glutamatergic neurons, including altered gene expression, defective cell connectivity, and abnormal cell morphology. This reveals that PRC2 plays a critical role in orchestrating mature neuronal identity and structure, suggesting its function extends beyond early progenitors (Currey et al. 2024). In *Drosophila*, loss of function of PRC2 core subunits (E(z), Su(z)12, esc) results in mild but significant defects in dendrite pruning, which is part of neuronal remodeling during metamorphosis (Bu et al. 2023). Moreover, the fragmentation and increased number of unpruned dendritic branches are more severe when both PRC2 and PRC1 are mutated (Bu et al. 2023). CRISPR/Cas9 knockout of Crooked legs (Crol), a transcription factor that recruits PcG in *Drosophila*, affects gene transcription (Erokhin et al. 2023). Alteration of H3K27me3 affects the temporal regulation of gene expression in developing postmitotic neurons, suggesting how H3K27me3 orchestrates temporal gene expression programs in neurogenesis (Ramesh et al. 2023).

## 9 HAT/ CBP/p300 Family

Histone acetyltransferases (HATs) are enzymes that govern epigenetic modification by catalyzing the transfer of an acyl moiety to lysine residues on the N‐terminal tails of histones. Histone acetylation is characterized by its rapid and reversible nature, and the modification is highly dynamic, underlying experience‐dependent behavioral changes (Mai et al. 2025). HATs are categorized into distinct families according to substrate preference, function, and sequence similarity; among them, CBP/p300, MYST, and GCN5/PCAF are the three most prominent HAT families, each playing a substantial role in brain function and cognition (Roth et al. 2001). p300 (also known as EP300 or KAT3b) and CBP (CREB‐binding protein or KAT3A) are the most well‐characterized; histone H3K9 residues are acetylated by p300, whereas H3K27Ac is catalyzed by either p300 or CBP (Ogryzko et al. 1996; Marsh et al. 2025). The binding and enrichment of P300 at H3K9Ac and H3K27Ac are relevant to features of transcriptionally active promoters and enhancers. Acetylation modifies the chromatin state to a more relaxed form, which enhances neural gene expression and enables the synthesis of new synaptic proteins (Chen et al. 2019). A study revealed that TTK21, a specific activator of CBP/p300 conjugated with glucose‐derived carbon nanospheres (CSP), could trigger dendritic branching and extend long‐term memory (Smitha et al. 2024). p300/CBP KAT activity induces the activation of five critical genes: Tubb3 (immature neural marker), Neurod1 (CNS development), Snap25 (spine morphogenesis and plasticity), Camk2a (synaptic plasticity and LTP), and Scn2a (propagation of the action potential), suggesting key roles for p300/CBP in neuronal progenitor cells (Smitha et al. 2024). A clinical study in mice showed that pharmacological activation of CBP/p300 could promote the expression of regeneration‐associated genes and the growth of axons in chronic spinal cord injury (SCI) with severe neurological disability (Muller et al. 2022). Rapid loss of molecular identity in neurons resulted from the deletion of CBP and p300 in hippocampal neurons, revealing that CBP/p300 maintains mature neuronal identity through regulation of histone acetylation of cell type‐specific genes (Lipinski et al. 2020). In *Drosophila*, overexpression of nej (the orthologue of human CBP/p300 proteins) in the nervous system results in a 6%–15% increase in lifespan, deciphering that *Drosophila* lifespan is regulated by nejire gene expression (Koval et al. 2023). Taken together, the HAT family CBP/p300 is involved in neurogenesis in both mammals and *Drosophila*, shedding light on the potential of manipulating the neuroepigenome as a new strategy to tackle age‐related neurodegenerative disorders (NDs).

## 10 SWI/SNF (BAF) Complexes

The switch/sucrose nonfermentable (SWI/SNF) chromatin remodeling complex is a member of the ATP‐dependent chromatin remodelers. This complex was first identified in yeast and includes polybromo‐associated Brg1‐associated factors (PBAF), Brg1‐associated factors (BAF), and noncanonical BAF (the mammalian‐specific ncBAF) (Fountain and Sauka‐Spengler 2023). The SWI/SNF/BAF complex plays a pivotal role in facilitating chromatin accessibility for transcription factors, thereby regulating the transcriptional machinery at targeted genomic regions (Schuettengruber et al. 2017). Studies in mammals (BAFs, BRM‐associated factors) and insects (BAPs, brm‐associated proteins) have revealed that SWI/SNF complexes play a critical role in nervous system function (Chmykhalo et al. 2025). A study identifies BAF complex accessory factors (BCL7A), which are involved in lineage choice and differentiation of NPCs by regulating mitochondrial bioenergetics and Notch/Wnt pathway signaling, suggesting their pivotal role in the regulation of genes that potentiate mitochondrial OXPHOS (Wischhof et al. 2022). Another recent finding on human ESCs revealed that Brahma‐related gene 1 (BRG1) is essential for establishing the chromatin landscape of NPCs via lineage‐specific transcription factors. Thus, BRG1 ensures appropriate expression of lineage‐specific TFs and proper activation of transcriptional programs to mediate NPC specification (Hoffman et al. 2024).

Structural and genetic analyses across diverse datasets show that mSWI/SNF (BAF) complex mutations result in neurodevelopmental disorder (NDD) phenotypes (Valencia et al. 2023). The SWI/SNF complex of neurons (neuronal BRG1/BRM‐associated factor or nBAF) facilitates RNA Pol II pausing and signal‐dependent RNA Pol II recruitment (loading) and induces transcription of neuronal immediate early genes (IEGs). This reveals that the neuron‐specific nBAF complex is required for transcription by regulating aspects of RNA Pol II transcription and facilitating RNA Pol II elongation (Cornejo et al. 2024). Snr, a *Drosophila* orthologue of SMARCB1 (a SWI/SNF subunit), is required for proper NSC gene expression and for the transition of neuroepithelial to NB (Keegan et al. 2023). Loss of Snr1 in NECs causes the formation of premature NSC and loss in NSCs leads to inappropriate perdurance of NSCs into adulthood, suggesting SWI/SNF subunit is regulating differential gene expression in *Drosophila* neurogenesis (Keegan et al. 2023). Additionally, BAP‐specific subunit Osa loss leads to Notch ligand Delta hyperactivation and the development of ectopic sensory structures patterned in early development (Niederhuber et al. 2024). Osa is linked with the activation of some target enhancers and constraints of other target enhancers in the same cell (Niederhuber et al. 2024). Taken together, SWI/SNF (BAF) complexes play a significant role in neurogenesis, including NSC proliferation, neural fate and function, differentiation, and migration. Ablation or impairment of these complexes results in various neural perturbations.

## 11 Higher Order Chromatin Architecture During Neuronal Development

Chromatin compartmentalization, which comprises changes in chromatin spacing, chromatin density, or subunit composition, plays an important role in epigenome maintenance and regulation, which is correlated to neuronal differentiation processes (Clapier et al. 2017). Dynamic changes in 3D organization influence enhancer/promoter interactions during neuronal differentiation in *Drosophila* (Chathoth and Zabet 2019; Nothof et al. 2022). Gene expression is regulated by physical contacts between genes and their regulatory regions. DNA modifications and transcription factor binding alter the chromatin state that controls brain functions and is related to the development of several neurological disorders (Harabula and Pombo 2021). DNA replication, repair, recombination, and transcription are maintained by the 3D nuclear space. The space occupied by chromosomes in the nucleus is referred to as the chromosome territory (Rajarajan et al. 2018), and chromosomes can be organized into compartments, topologically associating domains (TADs), and loop domains. Although structural dynamics can be observed on TADs, TAD boundaries are stable over many cell divisions and do not change in diverse cell types or lineages, confirming that most TAD boundaries are conserved, whereas new boundaries can appear at promoters of developmentally regulated genes (Dixon et al. 2012; Bonev et al. 2017; Hansen et al. 2018). At the time of ESC differentiation into neurons, TADs change their conformation, becoming wider and/or forming meta‐TADs (Rajarajan et al. 2018). Neuronal differentiation is associated with highly cell type‐specific 3D genome remodeling, suggesting that an “open” chromatin structure is crucial for transcription to maintain pluripotency and neuronal plasticity (Hu et al. 2020). On the other hand, chromatin loops are required for reprogramming of pluripotent cells. For instance, an enhancer–promoter loop within the human Oct4 locus (pluripotency transcription factor‐encoding gene) is required for the expression of endogenous pluripotency genes (H. Zhang et al. 2013).

Allele‐specific structures may play an important role in dysregulation of the 3D genome in imprinting disorders. Targeted correction of 3D genome abnormalities could therefore complement methylation‐editing‐based restoration of normal imprinting. Chromatin remodelers are also frequently mutated in cancers and implicated in aging (Kadoch and Crabtree 2015; Sen et al. 2016; Tan et al. 2021).

NDDs such as autism spectrum disorders and intellectual disability are caused by defects in chromatin remodeling enzymes. For example, the chromodomain helicase DNA‐binding protein 4 (Chd4), an ATP‐dependent chromatin remodeling gene, is mutated in syndromic intellectual disabilities mainly associated with macrocephaly, facial dysmorphisms, hearing loss, ventriculomegaly, hypogonadism, and various congenital anomalies including heart defects and bone fusions (Weiss et al. 2016; Alendar et al. 2020). This demonstrates that Chd4 suppresses interactions within contact domains in the developing brain, such as the shift in compartmentalization of these domains in the nucleus in conditional Chd4 knockout mice (Goodman et al. 2020). Thus, dysregulation of genome architecture may be a key mechanism by which mutations in chromatin regulators lead to neurodevelopmental cognitive disorders, including autism spectrum disorders and intellectual disability (Goodman et al. 2020).

**FIGURE 2:**
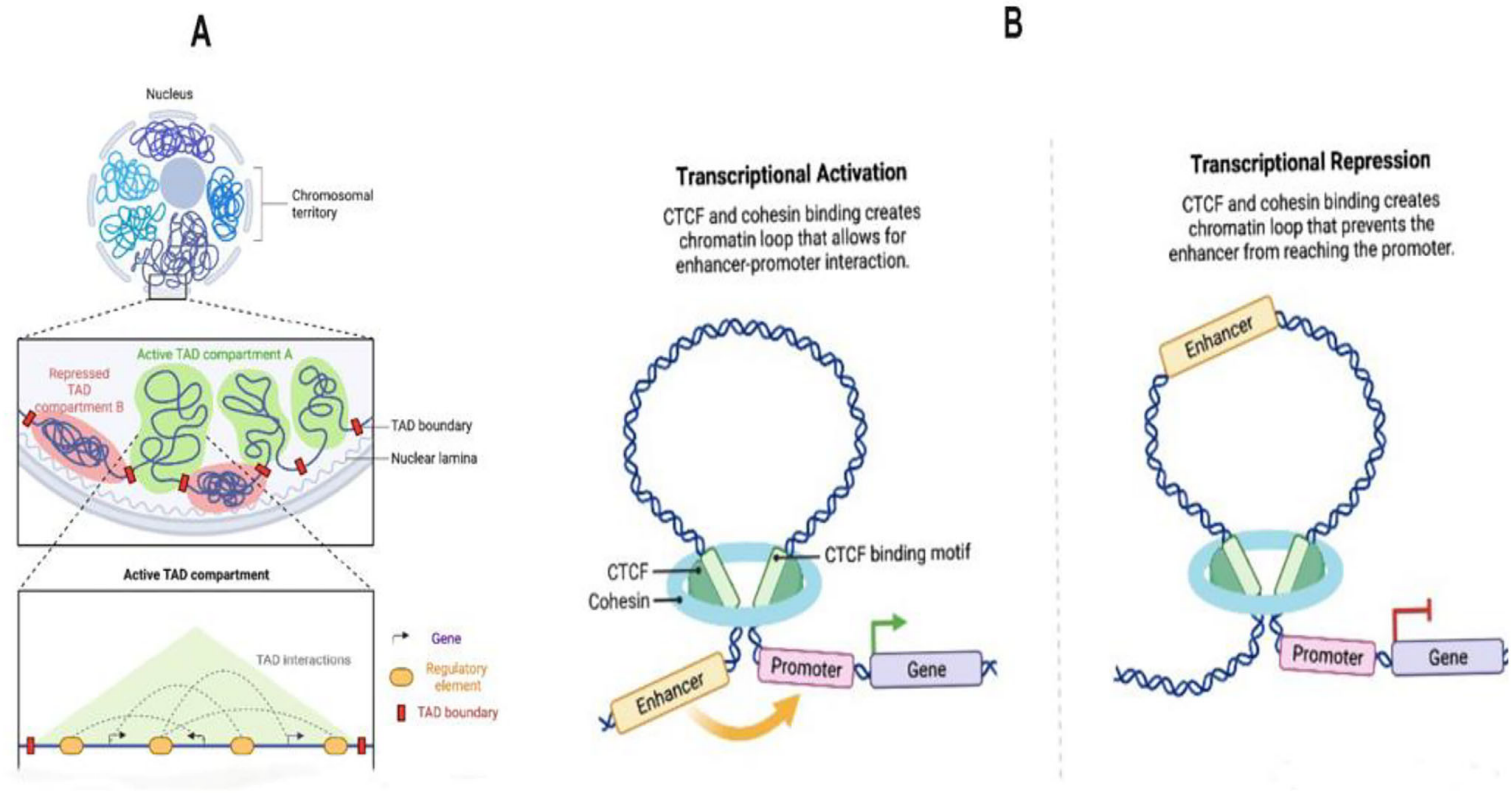
(A) Hierarchical organization of the genome in 3D space of the nucleus. (B) Transcriptional regulation by CTCF and cohesion (S. Kim et al. 2015).

Besides of local changes in chromatin state, the gene expression is also influenced by complex secondary and tertiary structures formed by chromatin folding and loop formation in the nucleus. Chromatin loops that form between promoters and enhancers cause expression of developmental genes (Figure 2). For instance, the chromatin loop controls the switch in globin gene expression during erythropoiesis (Rajarajan et al. 2019; Gallegos et al. 2018).

The protein meshwork known as the nuclear lamina, which is located at the inner layer of the nuclear membrane, plays a crucial role in cross‐linking with repressive chromatin regulators by tethering heterochromatic sequences to the nuclear periphery (Fujita and Yamashita 2018). In astrocytes, this interaction changes during neural progenitor cell differentiation as a result of the repositioning of many hundreds of genes during astrocyte differentiation (Camps et al. 2015; Peric‐Hupkes et al. 2010).

## 12 HDACs in Neurodegenerative Diseases

**FIGURE 3:**
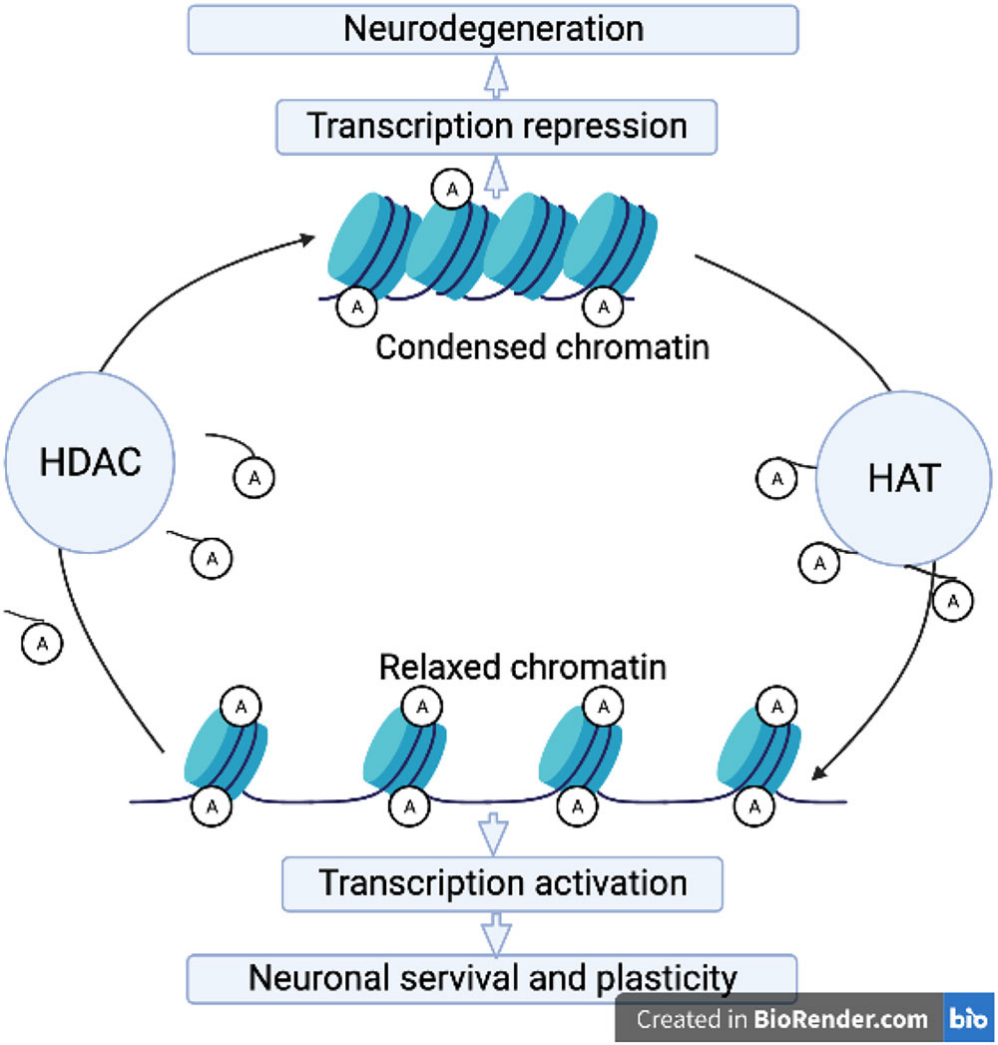
Schematic representation of the opposing functions of HATs and HDACs in regulating transcription activation and repression, and their downstream effects during neurogenesis.

The equilibrium activity of HDACs and HATs controls the acetylation and deacetylation of histone H3 and H4 proteins, which are the key epigenetic modifications controlling genes, during development and consequently in the development of neurodegenerative diseases and mental disorders (Cho and Cavalli 2014; Sharma et al. 2019; LoPresti 2020). Both HATs and HDACs are critical for brain development, and altered histone acetylation has been found in embryonic cortical neurons of some mouse models of cognitive defects (Penney and Tsai 2014; Sheikh 2014; X. Li, Egervari et al. 2018). HATs, which transfer acetyl groups from acetyl‐CoA to lysine residues, result in a loose chromatin state that allows transcriptional activation, whereas HDACs deacetylate lysine residues of histone proteins and remove the acetyl group from the histone tail, resulting in a tight, compact chromatin structure that suppresses transcriptional activity (Doke et al. 2021; Asfaha et al. 2019). In addition to lysine residues of histone proteins, both enzymes are also involved in the transfer and removal of the acetyl groups from 50 recognized nonhistone proteins. Several biological processes such as cell growth, cell death, and cellular signal transduction are influenced by HDACs (Gregoretti et al. 2004). Abnormal HDACs activity and overexpression result in NDs, cardiac diseases, cancer, HIV, diabetes, and inflammation, whereas inhibition of HDACs elevates the level of acetylated histones and other nonhistone proteins including signaling, mediators tumor suppressors, transcription factors, and DNA repair proteins (Marzio et al. 2000; Kook et al. 2003; Yuan et al. 2005; R. Wang et al. 2005; Gediya et al. 2021). Less is known about the therapeutic approach and prevention of disease progression in patients. Evidence in neurodegenerative diseases shows that HDAC is involved in neuronal apoptosis during the progression of these diseases (Gediya et al. 2021; Figure 3).

**FIGURE 4:**
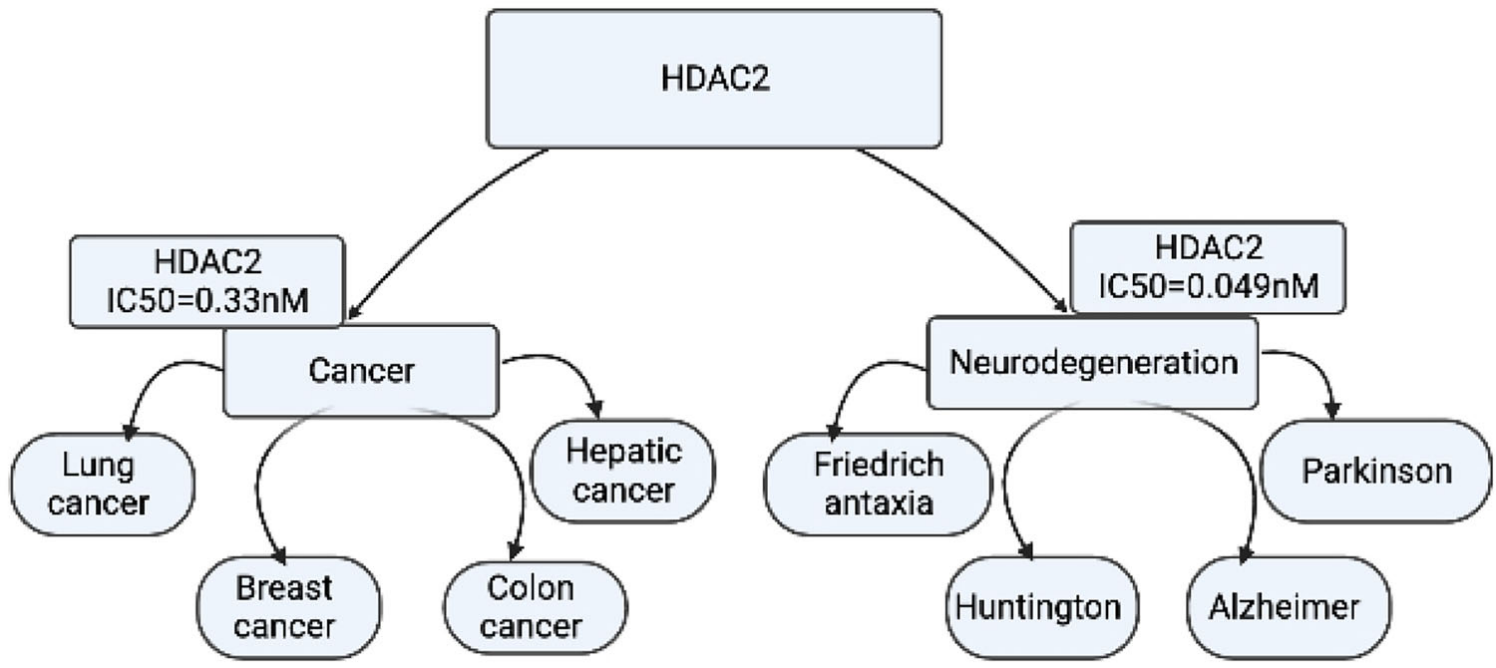
HDAC2 plays a pivotal role in epigenetic regulation and represents a prominent therapeutic target under investigation for both cancer and neurodegenerative disease treatment (modified from Gediya et al. [2021]).

Histone acetylation has been linked to synaptic plasticity and learning behavior through CREB–CBP‐dependent transcriptional activation (Vecsey et al. 2007). HDACs are a superfamily of enzymes that includes classes I, IIa, IIb, III, and IV. Class I (HDAC1, HDAC2, HDAC3, HDAC8) and class IIa (HDAC4, HDAC5, HDAC7, HDAC9) HDACs share structural homology and consist of zinc‐dependent enzymes, and among class I enzymes, HDAC1 and HDAC2 are associated with cell cycle genes. Genetic models in mice have shown that HDAC1 and HDAC2 influence brain function and differentially regulate subsets of activity‐regulated genes related to plasticity and memory; HDAC2 is expressed more abundantly in neurons, and it has a negative function in regulating memory formation (Guan et al. 2009; Figure 4). Another study has shown that DNA damage and cell death occur upon loss of HDAC1 function in neurons (D. Kim et al. 2008). A study in SH‐SY5Y cells showed that reduced HDACs promote histone acetylation, which likely contributes to neurodegeneration induced by Parkinson's disease (PD) neurotoxins (Lang et al. 2012). Deciphered protein expression of HDAC1, HDAC2, and HDAC6 is lower in PD midbrain samples than in the normal state (Park et al. 2016). Low expression of HDAC4 protein leads to more severe symptoms of brachydactyly mental retardation syndrome (BDMR), which is characterized by developmental delays, intellectual disability, behavioral abnormalities, and craniofacial and skeletal abnormalities. HDAC4 is also correlated with Huntington's disease (HTT) (Lang et al. 2012). HDAC9 is hemizygously deleted in schizophrenia patients and is also highly expressed in areas of the mouse brain associated with schizophrenia (Lang et al. 2012).

Moreover, targeting HDACs with HDAC inhibitors (HDACi) may have potential for treating neurological disorders such as HTT, AD, amyotrophic lateral sclerosis, seizure disorders, spinal muscular atrophy, Rett syndrome, stroke, Fragile X syndrome, and Rubinstein‐Taybi syndrome (Ziemka‐Nalecz et al. 2018; Siebzehnrübl et al. 2018). HDAC inhibition could provide a therapeutic avenue for memory impairment due to neurodegenerative and other diseases. It could also act through a hormesis effect by causing low‐dose damage that activates stress resistance, providing additional benefits to the organism (Vaidya et al. 2021). HDAC inhibitors may also directly act on and reverse age‐related changes or hallmarks of aging (epigenetic alterations, telomere attrition, genomic instability, loss of proteostasis, deregulated nutrient sensing, mitochondrial dysfunction, cellular senescence, stem cell exhaustion, and altered intercellular communication) (Vaidya et al. 2021). While preclinical studies indicate that HDAC inhibitors can reduce amyloid‐β and tau pathology, and restore cognitive function in mouse models of AD, human trials to date have been less conclusive. For example, a randomized, double‐blind, placebo‐controlled trial of valproate in mild‐to‐moderate AD (the VALID trial) failed to show benefit in delaying cognitive decline or institutional behavioral symptoms, and higher doses led to worsened cognition in some instances. Similarly, a phase II trial of the HDAC modulator FRM‐0334 in frontotemporal dementia, though safe, has not yet demonstrated clear clinical efficacy(Fessler et al. 2013).

## 13 Inhibition of HDAC for Therapeutic Intervention

HDACi are small natural or synthetic molecule that inhibit HDAC enzymes have a potentially therapeutic applications. For example, the common HDACi class such as hydroxamates, which specifically are pan inhibitors for class I‐II HDACs (trichostatin A [TSA], vorinostat, panobinostat), HDAC6 specific (tubastatin A and tubacin); cyclic peptides that specifically inhibit class I HDAC (romidepsin, apicidin, cyclic hydroxamic acid‐containing peptides [CHAPs]); aliphatic acids inhibits pan inhibitors for class I–II HDACs (butyrate, phenyl‐butyrate, valproate); benzamides specific to class I (MS‐275) (Mazzocchi et al. 2022; Govindarajan et al. 2013); sirtuin inhibitors are pan inhibitor (nicotinamide), SIRT2 specific (sirtinol, AK‐7, splitomicin); as well as panobinostat (LBH589) and TSA are nonspecific inhibitors of all the HDAC isoforms (Shukla and Tekwani 2020; Ziemka‐Nalecz et al. 2018;Didonna, and Opal 2015; Yoon and Eom 2016). However, a study has shown that molecule MC1568 inhibits specifically class IIa HDAC (Mazzocchi et al. 2022); administration of the class IIa HDI MC1568 exerts neuroprotection and behavioral improvements in a rat model of PD. Pharmacological studies show that complete knockout of HDAC6 leads to restore learning and memory in a severe AD model (APPPS1‐21 mice) by rescuing axonal transport (Govindarajan et al. 2013).

Moreover, targeting HDACs with HDACi might have potential for treatment of neurological disorders such as HTT, AD, amyotrophic lateral sclerosis, seizure disorders, spinal muscular atrophy, Rett syndrome, stroke, Fragile X syndrome, and Rubinstein‐Taybi syndrome (Ziemka‐Nalecz et al. 2018; Siebzehnrübl et al. 2018). HDAC inhibition may provide a therapeutic avenue for memory impairment caused by neurodegenerative and other diseases; it may also act through a hormesis effect, causing low‐dose damage that activates stress resistance, resulting in net benefit for the organism (Vaidya et al. 2021). HDAC inhibitors can also directly involve and reverse aging‐related changes or hallmarks of aging (epigenetic alterations, telomere attrition, genomic instability, loss of proteostasis, deregulated nutrient sensing, mitochondrial dysfunction, cellular senescence, stem cell exhaustion, and altered intercellular communication) (Vaidya et al. 2021).

## 14 Conclusions

Neurogenesis, the generation of diverse neuronal cell types together with neurulation and neuronal migration, underlies CNS development. During this process, the neuroepithelium, derived from the neural ectoderm, gives rise to neural progenitor cells (NPCs), which undergo tightly regulated proliferation and differentiation to produce neurons and glial cells. Perturbations in these processes can disrupt cell fate specification, maturation, and circuit formation, ultimately leading to neurodevelopmental and neurological disorders characterized by impairments in cognition, motor function, and behavior. Increasing evidence highlights epigenetic mechanisms, particularly HPTMs, as critical regulators of gene expression programs that govern neurogenesis. Histone methylation and acetylation, together with chromatin remodeling, orchestrate transcriptional regulation in a state‐dependent fashion to meet the dynamic demands of neural development. Henceforth, in this review, we have considered several mechanisms by which histone modifications and chromatin remodeling influence neurogenesis, which can be summarized as follows:

1. Histone methylation, particularly H3K9me2/me3 and H3K27me3, is essential for maintaining lineage fidelity, silencing developmental stage and tissue‐specific genes, and preserving genome stability within constitutive and facultative heterochromatin. Disruption of H3K9 histone methyltransferases such as G9a leads to impaired neural development, cell death, and developmental delay.
2. The dynamic and reversible interplay between histone methylation and acetylation at shared residues such as H3K9 represents a central regulatory mechanism in neurogenesis. Acetylation at H3K9 is generally associated with transcriptional activation, whereas methylation can confer repression. The reversible nature of these modifications allows chromatin to transition between permissive and repressive states, enabling precise temporal and spatial control of gene expression programs that regulate NPC proliferation, differentiation, and lineage commitment. The dynamic regulatory switch occurs on the same amino acid residue, enabling the regulation of gene expression during neurogenesis. Advanced cutting‐edge techniques including single‐cell epigenomics, which resolves modification patterns at the level of individual cells, and spatial chromatin profiling, which maps epigenetic features within their native tissue architecture, are set to reveal how dual H3K9 modification states correlate with cell identity, lineage progression, and pathological reprogramming in the nervous system.
3. Combinations of histone modifications, such as the H3 “K4me3‐K9me3” bivalent mark and its detection by ChIP‐Seq, are potentially functionally relevant for regulating clusters of genes, including rDNA genes, zinc finger proteins, long noncoding RNAs, and developmental signaling proteins. This bivalent combination of H3 “K4me3‐K9me3” methylation patterns plays a significant role in the recruitment of Spindlin1 and the maintenance of a “poised” chromatin state, suggesting that H3K9me3/2 exists not only in inactive gene regions but also co‐occurs with H3K4me2 in active gene regions, which may help to balance gene expression during neurogenesis.
4. Histone acetylation and deacetylation, regulated by HATs and HDACs, respectively, play critical and mechanistically complex roles in neurogenesis and neurodegeneration. HATs such as CBP/p300 promote chromatin opening and transcription of genes that support neuronal survival and plasticity, particularly in response to stress or injury, while distinct classes of HDACs regulate neural progenitor proliferation, differentiation, and synaptic function during development and adulthood. Dysregulation of HDAC expression and activity is linked to impaired neurogenesis and cognitive decline in neurodegenerative conditions, and pharmacological modulation of these enzymes has shown promise in ameliorating pathology and restoring function in neurodegenerative models. The balance of acetylation homeostasis is therefore a key determinant in neural development and disease, positioning both HATs and HDACs as pivotal regulators and therapeutic targets in brain disorders.
5. Chromatin remodeling complexes, including SWI/SNF, coordinate with histone modifications to regulate transcription by facilitating RNA polymerase II recruitment and/or progression, thereby supporting gene expression programs necessary for neurogenesis.

Despite progress made in the field, thorough in vivo investigations are limited due to the technical difficulties involved in monitoring neurons and isolating NSCs. Therefore, future studies should combine high‐resolution epigenomic profiling, single‐cell techniques, and advanced imaging methods to better understand the dynamic chromatin configurations during neurogenesis. Gaining these insights will enhance our comprehension of brain development and create new opportunities for therapeutic strategies that target epigenetic regulators in neurodevelopmental and neurological disorders.

## Author Contributions

D.Y.M. was involved in conceptualizing, drafting and writing the manuscript, and preparing figures; B.M.E. was involved in data curating, validating, and writing and editing the manuscript. All authors have commented, read, and approved the final manuscript as well as agreed to be accountable for all aspects of the work.

## Funding

This work was supported by the European Union‐Next Generation EU (Project 2022M75NN8, title “Dissection of common mechanisms in genetic primary microcephaly”; B53D23008280006; and B53D23008270006, funded to Laura Ciapponi and Maria Patrizia Somma).

## Ethics Statement

The authors have nothing to report.

## Conflicts of Interest

The authors declare no conflicts of interest.

## Data Availability

Data sharing is not applicable to this article as no new data were created or analyzed in this study.
